# Mortality Trends in Cancer of Unknown Primary Site in Serbia, 1990–2024: A Joinpoint Regression and Age–Period–Cohort Analysis

**DOI:** 10.3390/epidemiologia7020037

**Published:** 2026-03-02

**Authors:** Irena Ilic, Vladimir Jakovljevic, Milena Ilic

**Affiliations:** 1 Faculty of Medicine, University of Belgrade, 11000 Belgrade, Serbia; 2 Department of Physiology, Faculty of Medical Sciences, University of Kragujevac, 34000 Kragujevac, Serbia; 3 Department of Epidemiology, Faculty of Medical Sciences, University of Kragujevac, 34000 Kragujevac, Serbia

**Keywords:** cancer of unknown primary site, mortality, trends, joinpoint regression analysis, age–period–cohort analysis

## Abstract

Background/Objectives: Research on the mortality trends from cancer of unknown primary site in Serbia is scarce. This research sought to investigate temporal trends in mortality from cancer of unknown primary site in Serbia in the last few decades. Methods: This research was a population-based epidemiological descriptive study. Trends of age-standardized rates (ASRs) in mortality and average annual percent change (AAPC) were examined with joinpoint regression analysis. The age, period and cohort effects on mortality rates were evaluated using the age–period–cohort analysis. Results: From 1990 to 2024, 26,597 deaths from cancer of unknown primary site were registered in Serbia (14,944 in males and 11,613 in females). The trends for ASRs of mortality from cancer of unknown primary site in Serbia were stable for both males (AAPC = 0.2%, 95% CI = −0.4 to 0.7) and females (AAPC = 0.4%, 95% CI = −0.1 to 0.9) in all ages. Decreasing trends in mortality were observed in those under 55 years of age, while increasing trends were observed in older persons (55+), both in men and women. All estimated functions (period effect, cohort effect, the local drifts and net drift) for mortality in males in Serbia were significant (*p* < 0.05). For mortality in females, statistical significance was observed for cohort and period effects, along with the local drifts (*p* < 0.05 for all), while no statistical significance was observed for the net drift (*p* > 0.05). Conclusions: Mortality trends due to the cancer of unknown primary site were particularly unfavorable in elderly, suggesting that this burden of disease remains a public health issue in Serbia.

## 1. Introduction

Cancer of unknown primary (CUP) site refers to metastatic malignancies in which the origin cannot be identified during life, even after extensive clinical work-up [1,2,3]. Cancers of unknown primary site are a heterogeneous group of very aggressive unpredictable malignancies, with limited treatment options and very low overall survival [4,5]. The median survival in patients with CUP was around 3–5 months (with being men, older people, blacks, and having liver and pancreatic metastases increasing the risk of death), with approximately one fifth of patients alive at one year and 10% at five years [4,5,6]. In addition, the pattern of survival rates in patients with CUP has remained fairly consistent over the past few decades [7,8,9]. Among CUP, the most frequent histological type is adenocarcinoma (which accounts for almost 80% of all types), followed by undifferentiated carcinoma, squamous cell carcinoma and neuroendocrine carcinoma [10,11,12]. However, some recent research among selected patients has reported median survival times of 1 to 2 years, indicating some positive prognostic factors (including lymph node metastases, site-specific first-line therapy versus empiric chemotherapy, etc.) [13,14].

According to previous reports, CUP represents about 2–5% of all malignancies in both sexes [15,16]. CUP primarily affects older persons, with a median age of onset being approximately 60 years [14,17]. While CUP shows a higher prevalence among females, these findings are still not consistent [14,18,19].

Until now, the etiology of CUP remains unclear. Potential risk factors comprise older age, female sex, diabetes mellitus, smoking, obesity, alcohol consumption, food intake (animal or plant-based), physical activity, positive family history for cancer, personal medical history for some autoimmune diseases, lower socioeconomic status, and lower education level [20,21,22]. Socioeconomic differences in morbidity and mortality of patients with CUP have been observed in recent decades, which might be attributable to differences in the utilization of newer diagnostic techniques, availability of treatment modalities, and improved survival, but may also indicate differences in exposure to cancer risk factors (such as prevalence of smoking, diabetes, obesity, other lifestyle habits, etc.) and genetic factors between populations [15,23,24].

Mortality from CUP shows mixed trends over time and variation across countries: in many developed countries, trends increased or stabilized towards the end of the 20th century, and then declined [25,26,27]. In Northern Ireland, both male and female age-standardized mortality rates for CUP decreased in 2013–2022 (by 6.7% per year and by 5.3% per year, respectively), but those changes were not statistically significant [28]. Contrary to that, in developing countries (e.g., Serbia, India) there are very few reports on the epidemiology of CUP [29,30]. The present study sought to examine temporal trends in mortality from CUP site in Serbia across past decades.

## 2. Materials and Methods

### 2.1. Study Design

This population-based descriptive research with correlation study design examined data on mortality from CUP site in Serbia as the underlying death cause for the period 1990–2024. A completed REporting of studies Conducted using Observational Routinely-collected health Data (RECORD) Checklist [31] is provided in the Supplementary Materials.

### 2.2. Data Sources

Mortality data related to CUP site (International Classification of Diseases (ICD) to classify death, injury and cause of death revision 9 code 199 and revision 10 code C80) [32,33], that is “Malignant neoplasm without specification of site”, were retrieved from the Statistical Office of Serbia (unpublished data) [34]. Over the observed study period, main cause-of-death data in Serbia were coded using the ICD-9 during the 1990–1996 period, and from 1997 onward ICD-10 has been implemented [34].

Despite the literature showing an absence of a universally accepted definition, “cancer of unknown primary” (CUP) is clinically defined as a metastatic cancer confirmed by histology, for which the primary tumor is undetectable by a standardized diagnostic approach that includes detailed medical history assessment, complete physical examination (encompassing pelvic, breast, and rectal examinations, as well as imaging procedures such as chest X-ray, computed tomography of the abdomen and pelvis, mammography), and laboratory tests (including full blood count, biochemistry, urinalysis, stool occult blood testing and histopathological examination of biopsy material including immunohistochemical analysis with specific markers) [1,2,3].

In this study, deaths due to “Malignant neoplasm of other and ill-defined sites” (ICD-9 code 195; ICD-10 code C76), “Secondary and unspecified malignant neoplasm of lymph nodes” (ICD-9 code 196; ICD-10 code C77), “Secondary malignant neoplasm of respiratory and digestive organs” (ICD-9 code 197; ICD-10 code C78), and “Secondary malignant neoplasm of other sites” (ICD-9 code 198; ICD-10 code C79) were not included in analysis to minimize possible confounding [32,33].

According to Serbian law, a medical certificate of death is mandatory for all deaths. The determination of the time and cause of death is performed exclusively by a physician. For individuals who die within a healthcare institution where they have been treated, the certificate is issued by the healthcare facility, whereas for deaths occurring outside of medical institutions, the certification is carried out by a coroner, defined as a physician who has been given authority by the municipality to expertly establish the time and cause of death. Based on the guidelines of the World Health Organization (WHO), the underlying cause of death recorded on the medical certificate is defined as “the disease or injury which initiated the train of morbid events leading directly to death, or the circumstances of the accident or violence which produced the fatal injury” [34]. According to WHO assessments, mortality statistics in Serbia were rated as medium quality, with more than 90% completeness and fewer than 10% of deaths attributed to ill-defined causes [35].

The study covered the entire Serbian population, estimated at approximately 7.6 million in 1990 and around 6.6 million in 2024, according to censuses of 1991, 2002, 2011 and 2022, with intercensal estimates from the Statistical Office of the Republic of Serbia sourced for every other year in the studied period. Data on population of Serbia were sourced from the Statistical Office of Serbia (unpublished data) [34]. Over the research period, the population in Serbia experienced pronounced increasing retrograde trends in demography, including increased population decline (driven by increased mortality, decreased fertility, etc.), transitional trends in structure of population (marked population aging, etc.), intensified mobility (encompassing refugees, persons who are and internally displaced, young educated people emigrating abroad, etc.), prolonged economic downturn (unemployment, falling living standards, increasing poverty, etc.), and negative contextual factors (civil wars, country disintegration, international sanctions, NATO bombing) [34]. Over 1 million refugees resided in Serbia during the last decade of the 20th century, including around 500,000 in 1999 alone. At present, refugees are largely incorporated in the population of Serbia, precluding distinguishing refugee-specific data separately.

Data on measures that indicate level of human development, including Gross Domestic Product (GDP), GDP per capita and the Human Development Index (HDI), were obtained from the United Nations data for National Accounts Main Aggregates database [36], whereas information on the Socio-development Index (SDI) was sourced from the Global Burden of Disease study [37].

### 2.3. Study Variables

Mortality from cancer of unknown primary site was represented using age- and sex-specific as well as age-standardized rates (ASRs), expressed per 100,000 people. Age standardization was performed by direct method, using the World standard population.

The HDI is a composite measure of human development based on three indicators: average life expectancy (health), average and expected years of schooling (education), and standard of living (income) [36].

The SDI is a composite measure of human development reflecting income per capita, average educational attainment among persons aged 15 and older, and total fertility rate among women younger than 25 years [37]. Both HDI and SDI are scaled from 0 (lowest development) to 1 (highest development).

### 2.4. Statistical Analysis, Study Measures

#### 2.4.1. Statistical Analysis

The Joinpoint regression analysis (Joinpoint regression software, Version 4.9.0.0—March 2021, available via the Surveillance Research Program of the US National Cancer Institute) was used to examine temporal trends in mortality from cancer of unknown primary site, in accordance with the method proposed by Kim et al. [38]. Joinpoint regression analysis was used to identify the point (the so-called “joinpoint”) which depicts occurrence of a change in the trend which showed statistical significance. The Grid Search Method was employed [39]. In this analysis, the number of joinpoints applied as the minimum was zero, while five was applied as the maximum. The Monte Carlo Permutation method with 4499 randomly selected data sets was used [38]. The magnitude and direction of the trends in mortality in the 1990–2024 period were evaluated using the annual percent change (APC) and average annual percent change (AAPC) alongside the corresponding 95% confidence interval (95% CI) [40]. Also, a comparability test was conducted to identify if changes in mortality trends were parallel [41]. The analysis covered consecutive 10-year intervals, up to the age group of 85 years or older. The joinpoint regression analysis results were presented in a merged manner for all subgroups that are under 45 years, due to less than five deaths from CUP site occurring in each of the five-year periods in any year, thus making mortality rates unstable.

Additionally, the web-based statistical tool developed by the US National Cancer Institute was used to perform age–period–cohort analysis with the aim of investigating the effects of age, period and birth cohort on mortality trends, in accordance with the methodology described by Rosenberg et al. [42]. The analysis utilized mortality data grouped into consecutive five-year age groups (from 0–4 to 80–84), and five-year intervals for calendar periods (from 1990–1994 to 2020–2024) and birth cohorts (from 1905–1909 to 2020–2024). The results are presented in a merged manner for all subgroups aged 85+ years. Age–period–cohort analysis functions included period and cohort effects, and local drifts with net drift.

Further, linear regression models (measures by coefficient of determination, R2) were used to examine the link between the ASRs for mortality from CUP site and measures of socioeconomic and human development. The SPSS Software (version 20.0; Chicago, IL, USA) was used to conduct this analysis.

The computations (joinpoint analysis, age–period–cohort analysis and linear regression analysis) were performed by sexes. Statistical significance was considered at *p* < 0.05.

#### 2.4.2. Study Measures

Annual Percent Change (APC) measures trends in mortality rates, and represents the average rate of change per year over a specific time segment within a joinpoint regression model [40]. Average annual percent change (AAPC) is a summary measure that represents a weighted average of APCs over a fixed period which is pre-specified (in this study 1990–2024) [40]. Both APC and AAPC with the corresponding 95% confidence intervals (95% CIs) in mortality in the studied period were calculated. Trends were determined as “significant” (increasing, decreasing) according to significance of APC/AAPC compared to zero (*p* < 0.05), whereas trends were regarded as “stable” when APC/AAPC did not significantly differ from zero, with the 95% confidence interval including zero (*p* > 0.05).

Within the age–period–cohort method, all estimable functions were defined [42]. Period effects are changes in rates of mortality observed over time simultaneously affecting all age groups, whereas cohort effects represent variations in rates of mortality among groups of persons sharing same birth years. Annual percentage changes in mortality over studied time for each age group are local drifts, while net drift shows overall average annual percentage change in mortality during the period studied. The 1-df Wald test was used to determine significance.

The coefficient of determination (R2) is a statistical measure in linear regression that shows how much of the variability in the dependent variable can be explained by the independent variable in the model [43]. The values for R2 range from 0 to 1.

### 2.5. Ethical Considerations

The study was conducted according to the guidelines of the Declaration of Helsinki, and approved by the Ethics Committee of the Faculty of Medical Sciences, University of Kragujevac (Ref. No.: 01-14321 and date of approval 30 November 2017). The study was conducted using publicly available data (that is aggregated data, not individually identifiable).

## 3. Results

In the period from 1990 to 2024, 26,597 deaths from cancer of unknown primary location were registered in the population of Serbia (14,944 in men and 11,613 in women) (Table 1). The average ASR of mortality from cancer of unknown primary location was 5.10 per 100,000 in both sexes combined. The lowest average ASR of mortality (3.99 per 100,000) was registered during the 1990 to 1994 period, whereas the highest one (6.37 per 100,000) was from 2000 to 2004.

Concurrently, the average ASR of mortality from cancer of unknown primary location was 6.45 per 100,000 in males, and 3.94 per 100,000 in females. The lowest average ASR in mortality from cancer of unknown primary site for both males and females (5.09 per 100,000 and 3.08 per 100,000, respectively) was recorded in the period from 1990 to 1994, while the highest (8.32 per 100,000 and 4.72 per 100,000, respectively) was in the period from 2000 to 2004.

The average age-specific mortality rates from CUP site during the observed period increased markedly with age (Table 2). The mortality rate of this malignant disease was about 0.55 per 100,000 both in the male and female population younger than 45 years, whereas death rates around 90 times higher in men (about 58 per 100,000) and around 80 times higher in women (about 39 per 100,000) were recorded in the population aged 75 years or older.

**Figure 1:**
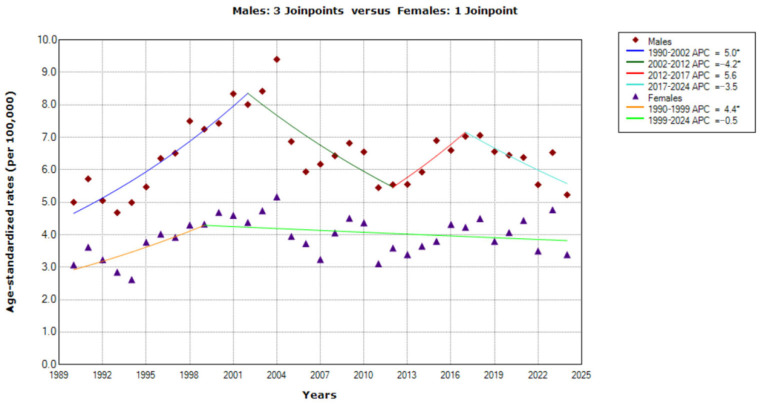
Trends in age-standardized rates (per 100,000) of mortality from cancer of unknown primary site in Serbia, 1990–2024 (single years), by sexes. * Statistically significant trend (*p* < 0.05); APC = Annual Percentage Change.

**Figure 2:**
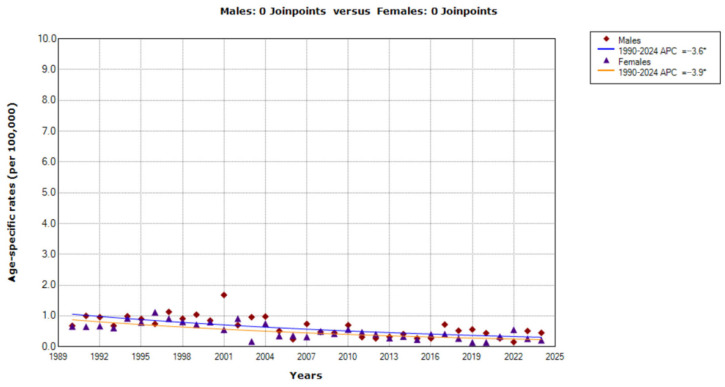
Trends in age-specific rates (per 100,000) of mortality from cancer of unknown primary site in Serbia, 1990–2024 (single years), by sexes (aged 0–44). * Statistically significant trend (*p* < 0.05); APC = Annual Percentage Change.

**Figure 3:**
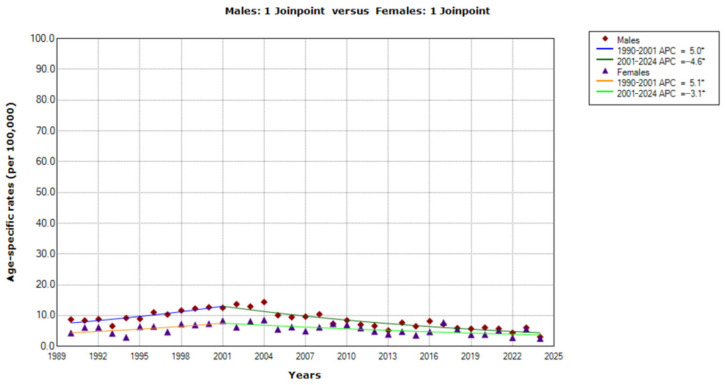
Trends in age-specific rates (per 100,000) of mortality from cancer of unknown primary site in Serbia, 1990–2024 (single years), by sexes (aged 45–54). * Statistically significant trend (*p* < 0.05); APC = Annual Percentage Change.

**Figure 4:**
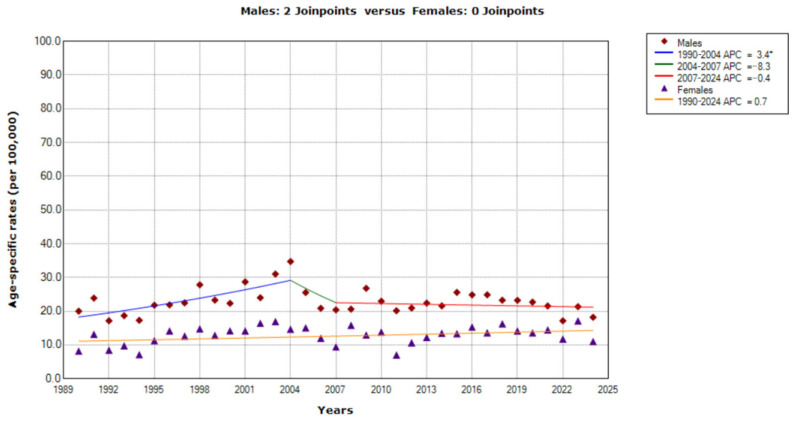
Trends in age-specific rates (per 100,000) of mortality from cancer of unknown primary site in Serbia, 1990–2024 (single years), by sexes (aged 55–64). * Statistically significant trend (*p* < 0.05); APC = Annual Percentage Change.

**Figure 5:**
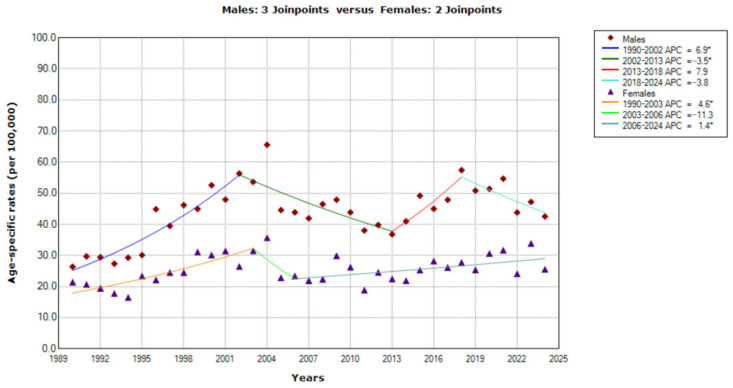
Trends in age-specific rates (per 100,000) of mortality from cancer of unknown primary site in Serbia, 1990–2024 (single years), by sexes (aged 65–74). * Statistically significant trend (*p* < 0.05); APC = Annual Percentage Change.

**Figure 6:**
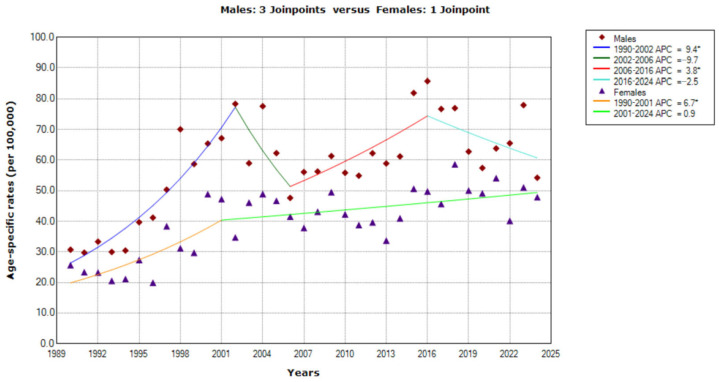
Trends in age-specific rates (per 100,000) of mortality from cancer of unknown primary site in Serbia, 1990–2024 (single years), by sexes (aged 75–84). * Statistically significant trend (*p* < 0.05); APC = Annual Percentage Change.

**Figure 7:**
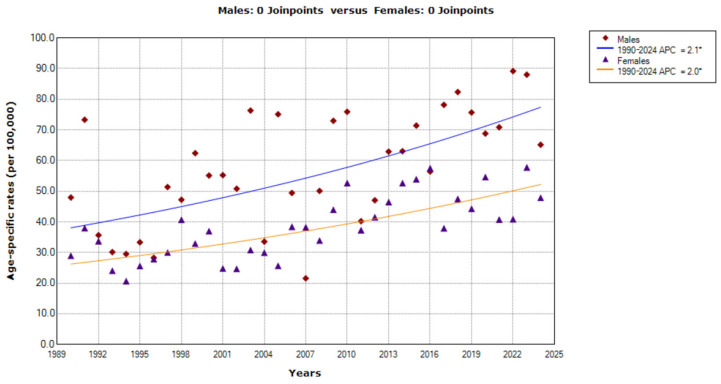
Trends in age-specific rates (per 100,000) for mortality from cancer of unknown primary site in Serbia, 1990–2024 (single years), by sexes (aged 85+). * Statistically significant trend (*p* < 0.05); APC = Annual Percentage Change.

The findings of joinpoint regression analysis of mortality rates from cancer of unknown primary site in Serbia in 1990–2024 are presented in Figure 1, Figure 2, Figure 3, Figure 4, Figure 5, Figure 6 and Figure 7. From 1990 to 2024, the trends in ASRs of deaths from CUP site in Serbia were stable for both males (AAPC = 0.2%, 95% CI = −0.4 to 0.7) and females (AAPC = 0.4%, 95% CI = −0.1 to 0.9) in all ages (Figure 1). Joinpoint regression analyses of mortality from cancer of unknown primary site in men identified three joinpoints (in 2002, 2012 and 2017), with four trends. The first and second period showed significant changes: a significantly increasing trend from 1990 to 2002, with APC = 5.0% (95% CI = 3.3 to 6.7), followed by a significantly decreasing trend from 2002 to 2012, with APC = −4.2% (95% CI = −6.6 to −1.7). The trend from 2012 was characterized by non-significant changes: a non-significantly increasing trend with APC = 5.6% (95% CI = −3.4 to 15.3) from 2012 to 2017, and a non-significantly decreasing trend from 2017 onwards, with APC = −3.5 (95% CI = −7.1 to 0.2). In females, joinpoint regression analysis identified a single joinpoint in 2009, with, consequently, two trends: a significantly increasing trend from 1990 to 1999, with APC = 4.4% (95% CI = 0.7 to 8.2), followed by a non-significantly decreasing trend from 1999 onwards, with APC = −0.5% (95% CI = −1.2 to 0.3). Throughout the study period, ASRs for mortality from cancer of unknown primary site in Serbia in females did not reach ASRs in males. The comparability test indicated that mortality trends for CUP site in men and women in Serbia during the observed period were parallel (final selected model: failed to reject parallelism; *p* = 0.343).

Decreasing trends in mortality from CUP site were recorded in the youngest age group (0–44 aged) during the entire period in both sexes (AAPC = −3.6%, 95% CI= −4.9 to −2.2 in males; AAPC = −3.9%, 95% CI = −5.1 to −2.6 in females) (Figure 2). The comparability test indicated that those mortality trends were parallel (*p* = 0.804).

Among the middle-aged group (45–54 years), the trends in mortality from CUP site in Serbia showed a significant decrease for both men (AAPC = −2.3%, 95% CI = −3.1 to −1.4) and women (AAPC = −1.1%, 95% CI = −2.1 to −0.1) (Figure 3). In males, there was one joinpoint in 2001, as identified by the joinpoint regression analysis, with, consequently, two trends: a significantly increasing trend from 1990 to 2001 (AAPC = 5.0%, 95% CI = 1.8 to 8.4) followed by a significantly decreasing trend from 2001 to 2024 (AAPC = −4.6%, 95% CI = −5.6 to −3.6).

In females, there was one joinpoint in 2001, as identified by the joinpoint regression analysis, with, consequently, two trends: a significantly increasing trend from 1990 to 2001 (AAPC = 5.1%, 95% CI = 0.1 to 10.4) followed by a significantly decreasing trend from 2001 to 2024 (AAPC = −3.1%, 95% CI = −4.6 to −1.5). The comparability test indicated that those trends of mortality were not parallel (final selected model: rejected parallelism; *p* = 0.004).

Among the middle-aged group (55–64 years), the trend in mortality from CUP site in Serbia was stable among men (AAPC = −0.0%, 95% CI = −0.6 to 0.5), while joinpoint regression analysis found two joinpoints (in 2004 and 2007), with consequently three trends (Figure 4). First, a significantly increasing trend was observed from 1990 to 2004 (with APC = 3.4%, 95% CI = 1.7 to 5.1), followed by a non-significantly decreasing trend from 2004 to 2007 (with APC = −8.3%, 95% CI = −35.1 to 29.6), and then followed by a non-significantly decreasing trend from 2007 onwards (with APC = −0.4%, 95% CI = −1.6 to 0.9).

An increasing trend in mortality from CUP site among women in the same age group that was recorded over the entire observed period (AAPC = 0.7%, 95% CI= −0.0 to 1.5) has not reached statistical significance (*p* = 0.053).

Based on the comparability test, trends for mortality from cancer of unknown primary site in men and women were parallel (*p* = 0.208) among the middle-aged group (55–64 years) during the observed period.

Among elderly people (65–74 years), trends in mortality from CUP site in Serbia were significantly increasing over the considered period in both men (AAPC = 1.2%, 95% CI = 0.6 to 1.9) and women (AAPC = 0.8%, 95% CI = 0.2 to 1.3) (Figure 5).

The joinpoint regression analyses of mortality from cancer of unknown primary site in males identified three joinpoints (in 2002, 2013 and 2018), with four temporal trends. The first and second period demonstrated significant changes: a significantly increasing trend from 1990 to 2002, with APC = 6.9% (95% CI = 5.0 to 8.8), followed by a significantly decreasing trend from 2002 to 2013, with APC = −3.5% (95% CI = −5.7 to −1.2). The trend from 2013 was characterized by non-significant changes: a trend which non-significantly increased by APC = 7.9% (95% CI = −1.9 to 18.6) from 2013 to 2018, with a non-significantly decreasing trend from 2018 onwards, with APC = −3.8 (95% CI = −8.6 to 1.2).

In females, two joinpoints (in 2003 and 2006) were identified by the joinpoint regression analysis, with three temporal trends: a significantly increasing trend from 1990 to 2003 (with APC = 4.6%, 95% CI = 2.6 to 6.7), followed by a non-significantly decreasing trend from 2003 to 2006 (with APC = −11.3%, 95% CI = −39.3 to 29.5), and then followed by a significantly increasing trend since 2006 onwards (with APC = 1.4%, 95% CI = 0.2 to 2.7).

Based on the comparability test, the trends of mortality from cancer of unknown primary site in men and women were parallel (*p* = 0.159) among older (65–74 years) persons during the observed period.

Among old people (75–84 years), the trends in mortality from CUP site in Serbia were significantly increasing in both males (AAPC = 2.0%, 95% CI = 1.2 to 2.8) and females (AAPC = 2.3%, 95% CI = 1.6 to 3.0) (Figure 6).

Joinpoint regression analyses of mortality from cancer of unknown primary site in males identified three joinpoints (in 2002, 2006 and 2016), consequently with four trends. First, a significantly increasing trend was observed from 1990 to 2002, with APC = 9.4% (95% CI = 7.0 to 11.8), then a non-significantly decreasing trend from 2002 to 2006, with APC = −9.7% (95% CI = −25.0 to 8.8). The trend since 2006 showed a significantly increasing trend with APC = 3.8% (95% CI = 0.3 to 7.4) from 2006 to 2016, and with a non-significantly decreasing trend from 2016 onwards, with APC = −2.5 (95% CI = −6.4 to 1.5).

In females, one joinpoint (in 2001) was identified by the joinpoint regression analysis, with, consequently, two trends: a significantly increasing trend from 1990 to 2001 (with APC = 6.7%, 95% CI = 3.4 to 10.1), followed by a non-significantly decreasing trend from 2001 to 2024 (with APC = 0.9%, 95% CI = −0.2 to 1.9).

Based on the comparability test, the trends for mortality from CUP site in men and women were parallel (*p* = 0.291) among old people (75–84 years) during the observed period.

Increasing trends in mortality from CUP site were recorded among the oldest persons (85+ years) throughout the whole studied period in both sexes (AAPC = 2.1%, 95% CI= 1.1 to 3.2 in men; AAPC = 2.0%, 95% CI = 1.4 to 2.7 in women) (Figure 7). The comparability test indicated that those trends were parallel (*p* = 0.876).

**Figure 8:**
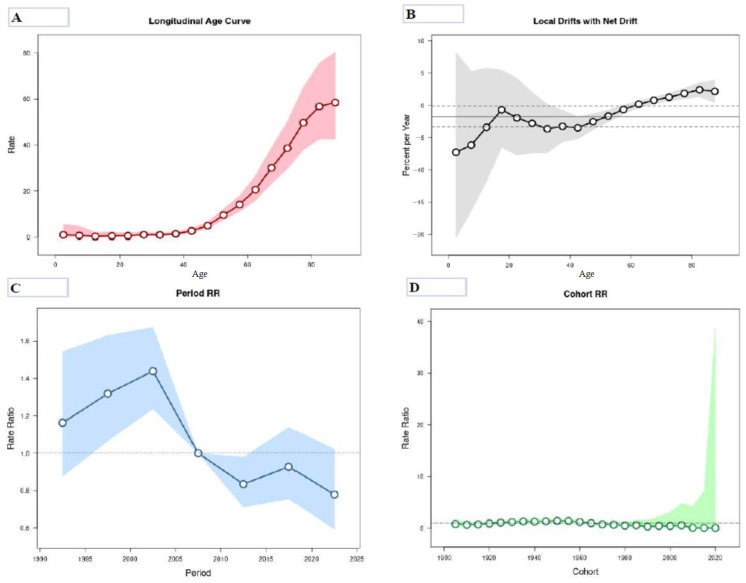
Mortality from cancer of unknown primary site in males in Serbia, 1990–2024, by age–period–cohort analysis. (**A**) Longitudinal age curve of cancer mortality rates (per 100,000 people) and corresponding 95% confidence intervals (pink area); (**B**) local drift value for cancer mortality rates: age group-specific annual percent change (%) in the cancer mortality rates and corresponding 95% confidence intervals (gray area); (**C**) period effects on cancer mortality rates: obtained from age–period–cohort analyses for cancer mortality rates and corresponding 95% confidence intervals (blue area); (**D**) cohort effects on cancer mortality rates: obtained from age–period–cohort analyses for the cancer mortality rates and corresponding 95% confidence intervals (green area). Source: Statistical Office of the Republic of Serbia [34].

**Figure 9:**
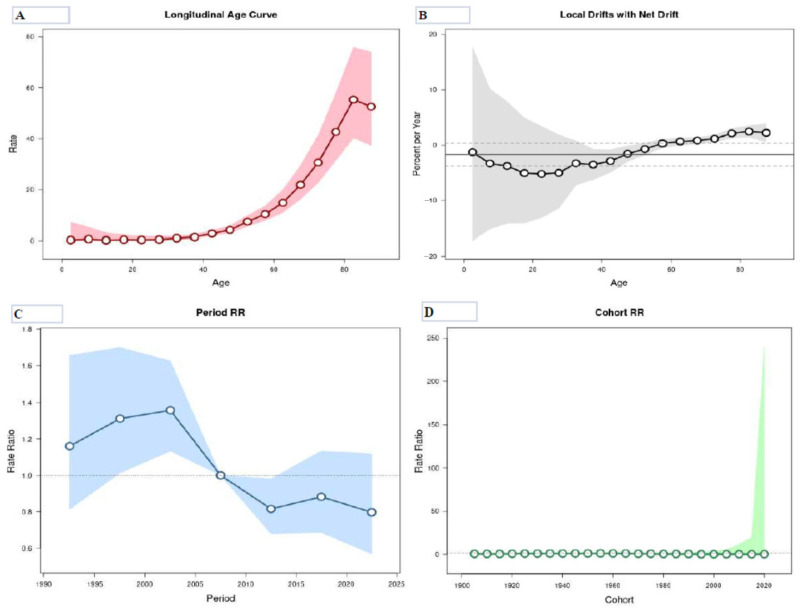
Mortality from cancer of unknown primary site in females in Serbia, 1990–2024, by age–period–cohort analysis. (**A**) Longitudinal age curve of cancer mortality rates (per 100,000 people) and corresponding 95% confidence intervals (pink area); (**B**) local drift value for cancer mortality rates: age group-specific annual percent change (%) in the cancer mortality rates and corresponding 95% confidence intervals (gray area); (**C**) period effects on cancer mortality rates: obtained from age–period–cohort analyses for cancer mortality rates and corresponding 95% confidence intervals (blue area); (**D**) cohort effects on cancer mortality rates: obtained from age–period–cohort analyses for the cancer mortality rates and corresponding 95% confidence intervals (green area). Source: Statistical Office of the Republic of Serbia [34].

The age–period–cohort analysis results regarding mortality from CUP site in men and women in Serbia from 1990 to 2024 are displayed in Table 3 and Figure 8 and Figure 9. The mortality risk from CUP site in Serbia was low for people younger than 40 in both sexes, with a few insignificant exceptions observed in particular in males.

Following the adjustment for period effects and for cohort effects, the risk of death from cancer of unknown primary site continuously increased after the age of 40 in both sexes: while the risk in males increased continuously with age, the risk in females reached its highest rates in people aged 80–84 and decreased thereafter. The risk of death was higher in men across all age groups, with the only exception in the 40–44 age group, when the rates in women slightly exceeded the rates in men. In the age group of 80–84, the relative risk of mortality was 20 times that observed in the 40–44 years in both sexes.

For mortality among men, the net drift was −1.78% (95% CI= −3.32 to −0.12) per year: the curves of local drift values were below 0 throughout all age groups under 60 (but showing a statistically significant increase only at the age groups 35–54 years), and then above 0 across all age groups over 64 years with a statistically significant elevation. For mortality in women, the net drift was −1.72% (95% CI= −3.75 to 0.35) per year: the curves of local drift values were below 0 throughout all age groups under 55 (but showing a statistically significant increase only in the age groups 35–50 years), and then above 0 across all age groups over 64 years with a statistically significant elevation. Although in both men and women the curve of local drift overlapped 0 in birth cohorts 65–69, in men the curve of local drift approached zero at a younger age (birth cohort 15–19) dividing the two statistically non-significant inflections observed in birth cohorts 0–14 and 20–59.

The period effects showed similar patterns in both sexes, which showed significantly elevated trends in 1995–2004 (compared with 2005–2009), followed by a slight decrease after, and then showed downward trends since 2005 with a significantly descending risk for the period 2010–2014. Also, the downward trends slowed after 2014 and shifted slightly upwards in 2015–2019.

The cohort effects on cancer of unknown primary site remained stable across the first several birth cohorts among males (for males born from 1905 to 1934). The risk of death in males increased for birth cohorts from 1935 to 1959 (compared with 1965–1969). The cohort effects descended from 1960 onwards (although most of these values were about 0).

The cohort effects on CUP site remained stable for the majority of birth cohorts among females. In contrast to males, birth cohort effects in females declined in birth cohorts 1910–1924 (compared with 1965–1969).

The age–period–cohort analysis showed that, in males, all estimated functions, i.e., period effect, cohort effect, local drifts and net drift for mortality from cancer of unknown primary site in Serbia, were statistically significant (*p* < 0.05). For mortality among females, Wald tests showed statistically significant cohort effects and period effects, and also local drifts (*p* < 0.05 for all), while the net drift did not show significance (*p* > 0.05).

**Figure 10:**
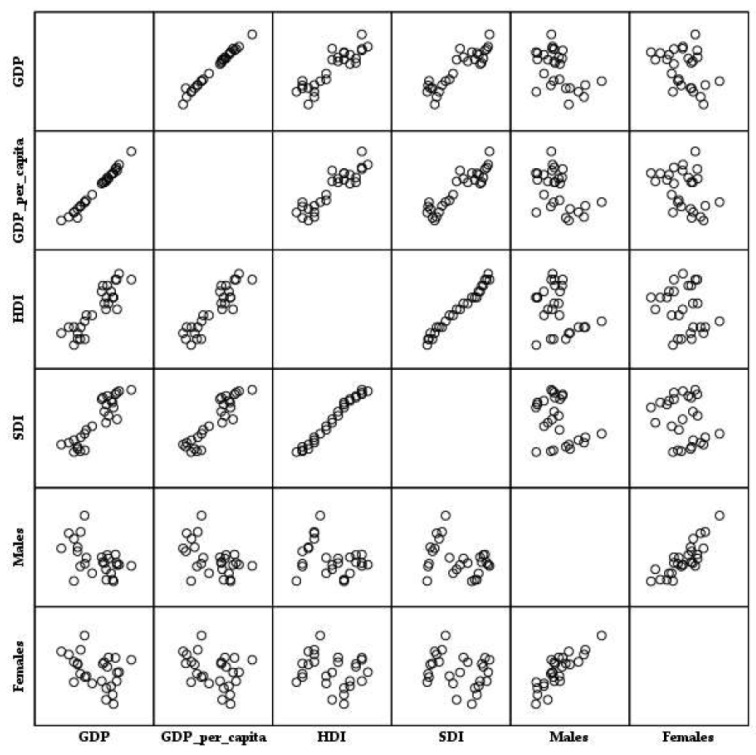
Association of the age-standardized mortality rates (per 100,000) from cancer of unknown primary site in Serbia with Gross Domestic Product (GDP), GDP per capita, Human Development Index (HDI), and Sociodemographic Index (SDI), by sexes, in 1990–2024. Data sources: Statistical Office of the Republic of Serbia [34], United Nations [36], and Institute for Health Metrics and Evaluation—Global Burden of Disease study [37].

Based on the results of the linear regression analyses, the ASR mortality rates (per 100,000) for CUP site among males in Serbia were significantly positively associated with GDP and GDP per capita in the 1990–2024 period (R2 = 0.337, *p* = 0.002; R2 = 0.292, *p* = 0.002, respectively), while there was an absence of association with HDI and SDI (R2 = 0.007, *p* = 0.631; R2 = 0.002, *p* = 0.809, respectively) (Figure 10).

Based on the results of the linear regression analyses, the ASR mortality rates (per 100,000) for CUP site among females in Serbia were not associated with GDP, GDP per capita, HDI and SDI in the 1990–2024 period (R2 = 0.107, *p* = 0.077; R2 = 0.096, *p* = 0.096; R2 = 0.008, *p* = 0.598; R2 = 0.019, *p* = 0.425, respectively).

## 4. Discussion

This comprehensive analysis of national mortality trends from cancer of unknown primary site in Serbia between 1990 and 2024 reveals complex temporal changes and substantial sex and age disparities. Over the considered period, the overall ASR of mortality from cancer of unknown primary site was 5.10 per 100,000, with higher rates in males than females. Age-specific analysis showed that mortality rates consistently increased with increasing age.

In our study, overall, the trends in ASRs of mortality from cancer of unknown primary site were stable for both males and females over the considered period of 1990–2024. The lowest ASRs of mortality from cancer of unknown primary site were observed during the 1990–1994 period, while the highest rates were recorded between 2000 and 2004. Contrary to this, many locations experienced notable declines in mortality rates over time [26,28,44,45]. These findings potentially suggest changes in diagnostic practices and cancer registration completeness or quality in Serbia. There is evidence that deprivation of individuals is associated with increased risk of cancer of unknown primary site [46]. In addition, research shows that improved overall survival in patients with CUP site is seen in those with higher education [15]. Lower educational attainment, as one component of the indicators of socioeconomic development, was confirmed as a factor associated with occurrence of cancer of unknown primary site [23], which could be related to low health literacy. The latest data available from the Republic of Serbia’s Statistical Office show that only 22.24% of population aged 15 and over had high and higher education in 2022 [47]. Countries with higher level of socioeconomic development likely have more developed healthcare systems, which are all closely related to access to healthcare and especially relevant in aging populations [48].

Males consistently exhibited higher mortality rates from cancer of unknown primary site compared to females, aligning with patterns reported in other populations [25,26,28,44,45]. Female sex has consistently been identified as a factor associated with improved survival in patients who have CUP site. Studies in the USA using data over the 1973–2008 period [15] and Switzerland covering the 1981–2014 period [6] showed that females had better survival compared to males, with HR around 0.86. A study in South Korea which included a shorter observation period of 1999–2017 also confirmed the pattern of higher survival rates in women [49]. These sex-specific differences in mortality could likely reflect differences in biology, healthcare utilization and variations in exposure to risk factors. The observed consistently higher mortality rates in men could reflect higher prevalence of smoking and alcohol consumption, and occupational exposures [20,50]. Additionally, research shows that men exhibit delayed health-seeking behavior compared to women [51]. Finally, the changes in survival could at least to some extent be explained by changes in survival rates of cancers which are known to often metastasize [52].

In this study in Serbia, linear regression analyses showed some discrepancies by sexes: although a significant positive association of mortality rates from cancer of unknown primary site with GDP and GDP per capita was reported in males in the period of 1990–2024, in females an absence of a significant association of mortality rates from cancer of unknown primary site with GDP and GDP per capita was reported, while in both sexes absence of an association with HDI and SDI was noted. The described findings run contrary to the results found in a previous study on a similar topic in Serbia [29], which inversely links higher socioeconomic indicators with improved CUP outcome. Differences between these two studies in Serbia could be attributed to differences in the ICD codes included in research (ICD codes C77-C80 vs. ICD80 code in the current study), different size of the population (Central Serbia vs. the Republic of Serbia entire in the current study), length of the observed period (1999–2018 vs. 1990–2024 in the current study), differences in the considered indicators of human development (only HDI as a summary measure vs. four measures including single measures such as GDP and GDP per capita, and summary measures such as SDI and HDI). In the current study, the overall trends in ASRs of deaths from CUP in Serbia were stable for both males and females in all ages together, and were parallel, with a significant increase at the beginning of the observed period (by 5.0% per year in males from 1990 to 2002, and by 4.4% per year in females from 1990 to 1999); this suggests that those trends cannot be explained by biological factors or lifestyle habits. Namely, the unfavorable period effects on CUP mortality that are similar both in males and females during the period 1990–2004 could probably be linked with adverse conditions associated with sociopolitical fluctuations in the country during the observed period, including the devastating consequences of the civil wars that occurred in the former Yugoslavia from 1991 to 1995, economic and political UN-imposed sanctions between 1992 and 1995, hyperinflation of the national currency in 1993, and the NATO-led bombing campaign in 1999, followed by a large-scale influx of over 500,000 refugees who needed care and imposed burden on economy at the national level, and broadly disrupted healthcare system (with a shortage of medicines and medical equipment, substantial increase in the number of wounded citizens and refugees who needed medical and social care, decrease in hospitalization rates, especially for person over 60 years of age) [53,54]. In the following years, the decreased period effects on CUP mortality may reflect economic progress and enhancements in overall living conditions, greater utilization of diagnostic techniques, improved treatment, screening testing, or lower exposures to risk factors for CUP (including smoking rates, alcohol use, etc.) due to implementation of national strategies for cancer prevention and management in Serbia. However, in contrast to the decrease in cohort effects on CUP mortality among the oldest females (in birth cohorts from 1910 to 1924), the current study showed an increase in the cohort effects on CUP mortality in middle- and older-aged males (in birth cohorts from 1930 to 1959) in Serbia. Significantly decreased cohort effects on CUP mortality in females in the oldest age groups and non-significantly decreased cohort effects in the same age groups in males could be related to differences in the epidemiological transition in relation to exposure to risk factors, primarily to the habit of smoking cigarettes, which was less prevalent in women than in men in Serbia in the first decades of the 20th century [55]. Although the precise mechanisms that could explain the positive association between mortality of CUP in males and GDP and GDP per capita in Serbia remain unknown, potential explanations may include factors related to occupation and environmental problems (including exposure to risk factors, e.g., potential consequences of bombardment with depleted uranium, clearing of terrain contaminated with toxic materials, etc.) [56,57], as well as the greater levels of responsibilities and stress experienced by men during periods of war and economic crises [58,59]. Cancer mortality as a consequence has been recorded in the history of warfare around the world [60]. However, since the national autopsy rates have generally been low (about 2%) in recent decades in Serbia [61], and since the percentage of histologically/cytologically confirmed cases was higher in males than in females (60.6% and 53.4%, respectively) [62], some uncertainty related to the presented findings on differences in association between CUP mortality with GDP and GDP per capita by sex cannot be completely excluded.

The joinpoint regression analysis identified more dynamic temporal changes among men, where mortality initially increased significantly from 1990 to 2002, declined significantly from 2002 to 2012 and then showed non-significant increase from 2012 to 2017 and non-significant decrease from 2017 onwards. In contrast, mortality trends in the ASRs of mortality from cancer of unknown primary location in females showed a significant increase from 1990 to 1999, followed by a non-significant decline towards the end of the observed period. According to the Cancer Research UK data, over the 1971–2023 period the ASRs of mortality from CUP in males as well as in females increased from 1973 to 1996–1998, and decreased afterwards, reaching a plateau from 2015–2017 onwards [26]. Contrary to this, in Northern Ireland the ASRs of mortality of unknown primary cancer have been decreasing from 2003 to 2022 both in men and women [28]. Similarly, the ASRs of mortality from CUP site in Australia decreased over the 1982–2023 period, from 15.6 and 11.7 deaths per 100,000 in males and females respectively, to 13.4 and 8.6 deaths per 100,000 in males and females respectively [44]. At the same time, South Australia experienced an increase in ASRs of mortality from cancer of unknown primary site from 1977–1980 to 1985–1988 calendar year period for both males and females, which then peaked in 1993–1996 and continued to decline to 2004 [25]. In Sweden, the overall survival among patients with CUP site significantly improved in the 1994–2000 period, with the highest improvement observed further on from 2001 to 2008, compared to 1987–1993 [63]. Compared to the mortality patterns in the above-mentioned research, Serbia has experienced a later decline in mortality from cancers of unknown primary site. This could be due to Serbia being a transitional economy, with incomplete access to advanced techniques in imaging, immunohistochemistry and molecular diagnostics [64]. Additionally, geographic variations could be attributed to non-unified definition and criteria for the diagnosis [14]. Furthermore, discrepancies could be due to changes in classification and coding of cancer. Finally, the issue of misclassification of causes of death and the lack of pathohistological data may lead to over- or under-estimation of mortality trends.

The age-specific analyses demonstrated mortality from cancer of unknown primary site to be clearly increasing with age. Trends in age-specific rates were significantly decreasing in age groups up to 54 years both in males and females; in those aged 55–64, the trend was stable in males and demonstrated an increase that was not significant in females, while in both males and females aged 65 and older the trends in age-specific rates were significantly increasing over the observed period. Similar findings of improved survival in younger age groups were observed in the Southeast Netherlands [65], the USA [15], Switzerland [6], Sweden [4] and Iran [66]. The decrease in age-specific rates observed in younger age groups could be due to earlier detection and improved diagnostic accuracy, and better access to specialized oncology care [67]. The stable and nearly unchanged trends in males and females aged 55–64 could possibly reflect persisting lifestyle risks [68,69]. Significantly increasing mortality rates in the oldest age groups could reflect demographic aging, improved case ascertainment and enhanced death certification practices.

Examining the mortality patterns in burden of CUP site is important for highlighting the areas where diagnostic efforts could be improved and where introduction of clinical guidelines for management based on the personalized medicine approach is necessary. Strengthening the efforts for targeted cancer control, especially for older adults, is needed.

### Strengths and Limitations of the Research

One of the main strengths of this research is the provision of comprehensive national estimates for mortality of cancer of unknown site in Serbia covering a 35-year period. Additionally, this study encompasses the entire Serbian population and uses quality cancer mortality data, with time trends assessed using both the joinpoint regression analysis and age, period and cohort analysis. In addition, this study provides correlation analysis mortality trends of cancer of unknown site with measures of human development and sociodemographic level in Serbia.

However, this study had several limitations. Variations in cancer mortality trends may reflect fluctuations in cause-of-death registration, and the reliability and validity of death certificates, which remain open to debate. However, the proportion of cases that have unclear causes of death did not decrease significantly throughout the research period, suggesting that a significant increase in mortality from cancer during the considered period can hardly reflect solely the improved mortality data quality in Serbia. The absence of separate cancer mortality data for refugees in Serbia may potentially distort the pattern of cancer mortality. In addition, the well-recognized limitations of the age–period–cohort analysis, including collinearity between age, period and cohort effects and ecological fallacy, should be acknowledged.

## 5. Conclusions

Although the trends in mortality from cancer of unknown primary site in Serbia were stable for both men and women in all ages over the last few decades, increasing trends were recorded in older persons both in men and women. While the period effect, cohort effect, local drifts and net drift for mortality in males in Serbia were significant, for mortality in females statistical significance was observed for cohort and period effects, and the local drifts, while the net drift was non-significant. Unfavorable mortality trends due to cancer of unknown primary site suggest that this health burden remains a public health issue in Serbia. Further analytical epidemiological research is needed for a clearer insight into the factors that contributed to changes in trends in order to improve understanding and determine strategies for more effective prevention and control of cancer.

## Figures and Tables

**Table 1:** Number and average age-standardized rates (ASRs, per 100,000) of mortality from cancer of unknown primary site in Serbia according to 5-year time period, by sexes.

	Both Sexes		Males		Females	
Time Period	No. of Deaths	Average ASR	No. of Deaths	Average ASR	No. of Deaths	Average ASR
1990–1994	2425	3.99	1395	5.09	1030	3.08
1995–1999	3415	5.22	1949	6.62	1466	4.07
2000–2004	4493	6.37	2598	8.32	1855	4.72
2005–2009	3800	5.06	2123	6.45	1677	3.90
2010–2014	3658	4.57	2036	5.80	1622	3.62
2015–2019	4534	5.55	2547	6.83	1987	4.13
2020–2024	4272	4.93	2296	6.03	1976	4.03
Total	26,597	5.10	14,944	6.45	11,613	3.94

**Table 2:** Average age-specific mortality rates (per 100,000) from cancer of unknown primary site in Serbia, 1990–2024, by sexes.

	Mortality Rates (95% CI)			
Age Group, Years	Males		Females	
0–44	0.65	(0.54–0.76)	0.51	(0.43–0.60)
45–54	8.65	(7.69–9.61)	5.62	(5.06–6.17)
55–64	22.85	(22.55–24.15)	12.91	(12.00–13.83)
65–74	43.94	(40.80–47.07)	25.45	(23.84–27.03)
75–84	58.32	(52.97–63.66)	39.93	(36.26–43.59)
85+	57.56	(51.22–63.90)	38.38	(34.82–41.96)

**Table 3:** Age, period and cohort effects on mortality from cancer of unknown primary site in Serbia, 1990–2024, by sexes.

		Males		Females	
		Effect	95% CI	Effect	95% CI
Age	0–4	1.01	0.19–5.52	0.29	0.01–7.16
	5–9	0.73	0.11–4.79	0.62	0.07–5.22
	10–14	0.36	0.06–2.13	0.20	0.01–3.04
	15–19	0.55	0.14–2.14	0.42	0.08–2.24
	20–24	0.61	0.20–1.19	0.33	0.06–1.72
	25–29	1.06	0.50–2.24	0.45	0.12–1.66
	30–34	0.98	0.51–1.89	0.97	0.50–1.88
	35–39	1.41	0.88–2.25	1.41	0.85–2.34
	40–44	2.69	1.90–3.82	2.72	1.84–4.03
	45–49	4.92	3.67–6.58	4.10	2.94–5.71
	50–54	9.53	7.41–12.25	7.32	5.49–9.76
	55–59	14.11	11.08–17.97	10.30	7.83–13.53
	60–64	20.59	15.89–26.69	14.74	10.94–19.88
	65–69	30.06	23.20–38.95	21.82	16.19–29.41
	70–74	38.61	29.69–50.21	30.55	22.61–41.29
	75–79	49.68	37.91–65.11	42.63	31.39–57.89
	80–84	56.75	42.58–75.63	55.29	40.27–75.90
	85+	58.44	42.48–80.40	52.55	37.31–74.01
Period					
	1990–1994	1.16	0.88–1.54	1.16	0.81–1.66
	1995–1999	1.32	1.07–1.63	1.31	1.01–1.70
	2000–2004	1.44	1.24–1.67	1.36	1.13–1.63
	2005–2009	1.00	1.00–1.00	1.00	1.00–1.00
	2010–2014	0.83	0.71–0.98	0.82	0.68–0.98
	2015–2019	0.93	0.76–1.14	0.88	0.69–1.14
	2020–2024	0.78	0.59–1.02	0.80	0.57–1.12
Cohort					
	1905–1909	0.82	0.38–1.75	0.60	0.29–1.26
	1910–1914	0.68	0.41–1.11	0.54	0.34–0.88
	1915–1919	0.75	0.50–1.13	0.49	0.31–0.76
	1920–1924	0.94	0.68–1.29	0.70	0.49–0.99
	1925–1929	1.11	0.83–1.47	0.88	0.64–1.21
	1930–1934	1.21	0.92–1.59	0.92	0.67–1.26
	1935–1939	1.33	1.02–1.73	0.97	0.72–1.32
	1940–1944	1.29	0.99–1.68	0.97	0.72–1.31
	1945–1949	1.33	1.03–1.74	1.01	0.75–1.37
	1950–1954	1.45	1.12–1.87	1.07	0.80–1.44
	1955–1959	1.41	1.09–1.83	1.15	0.86–1.55
	1960–1964	1.22	0.92–1.61	1.07	0.78–1.46
	1965–1969	1.00	1.00–1.00	1.00	1.00–1.00
	1970–1974	0.76	0.51–1.15	0.70	0.44–1.10
	1975–1979	0.70	0.41–1.17	0.67	0.37–1.21
	1980–1984	0.52	0.25–1.07	0.45	0.19–1.07
	1985–1989	0.60	0.22–1.64	0.44	0.14–1.36
	1990–1994	0.34	0.07–1.57	0.45	0.09–2.31
	1995–1999	0.48	0.10–2.34	0.14	0.01–2.31
	2000–2004	0.45	0.07–3.13	0.16	0.01–3.64
	2005–2009	0.59	0.07–4.78	0.17	0.01–5.66
	2010–2014	0.09	0.00–4.31	0.19	0.00–11.60
	2015–2019	0.07	0.00–7.19	0.15	0.00–19.87
	2020–2024	0.06	0.00–38.77	0.22	0.00–38.77
		Wald Chi-square tests for estimable functions, *p*-value			
Net drift		0.0351		0.1032	
All period rate ratios		<0.0001		<0.0001	
All cohort rate ratios		<0.0001		<0.0001	
All local drifts		<0.0001		0.0074	

95% CI = confidence interval. Source: Statistical Office of the Republic of Serbia [34].

## Data Availability

The underlying data is contained within the article and the cited data sources, whereas the unpublished data must be obtained directly from the Statistical Office of the Republic of Serbia under its terms of use.

## Supplementary Materials

The following supporting information can be downloaded at: https://www.mdpi.com/article/10.3390/epidemiologia7020037/s1, Table S1: The RECORD statement—checklist of items, extended from the STROBE statement, that should be reported in observational studies using routinely collected health data.
